# Cisplatin-resistance and aggressiveness are enhanced by a highly stable endothelin-converting enzyme-1c in lung cancer cells

**DOI:** 10.1186/s40659-024-00551-9

**Published:** 2024-10-24

**Authors:** Cristopher Almarza, Karla Villalobos-Nova, María A. Toro, Manuel González, Ignacio Niechi, David A. Brown‑Brown, Rodrigo A. López-Muñoz, Eduardo Silva-Pavez, Belén Gaete-Ramírez, Manuel Varas-Godoy, Verónica A. Burzio, Lilian Jara, Francisco Aguayo, Julio C. Tapia

**Affiliations:** 1 Programa de Biología Celular y Molecular, Instituto de Ciencias Biomédicas, Facultad de Medicina, Universidad de Chile, Santiago, Chile; 2 Instituto de Bioquímica y Microbiología, Facultad de Ciencias, Universidad Austral de Chile, Valdivia, Chile; 3 Instituto de Farmacología y Morfofisiología, Facultad de Ciencias Veterinarias, Universidad Austral de Chile, Valdivia, Chile; 4 Facultad de Odontología y Ciencias de la Rehabilitación, Universidad San Sebastián, Santiago, Chile; 5 Centro de Biología Celular y Biomedicina, Facultad de Medicina y Ciencia, Universidad San Sebastián, Santiago, Chile; 6 Departamento de Ciencias Biológicas, Facultad de Ciencias de la Vida, Facultad de Medicina, Universidad Andrés Bello, Santiago, Chile; 7 Programa de Genética, Instituto de Ciencias Biomédicas, Facultad de Medicina, Universidad de Chile, Santiago, Chile; 8 Departamento de Biomedicina, Facultad de Medicina, Universidad de Tarapacá, Arica, Chile; 9 Laboratorio de Transformación Celular, Programa de Biología Celular y Molecular, Instituto de Ciencias Biomédicas, Facultad de Medicina, Universidad de Chile, Av. Independencia 1027, Santiago, 8380453 Chile

**Keywords:** Endothelin, CK2, Ubiquitination, Stemness, Chemoresistance, Invasiveness

## Abstract

**Background:**

Lung cancer constitutes the leading cause of cancer mortality. High levels of endothelin-1 (ET-1), its cognate receptor ET_A_R and its activating enzyme, the endothelin-converting enzyme-1 (ECE-1), have been reported in several cancer types, including lung cancer. ECE-1 comprises four isoforms, which only differ in their cytoplasmic N-terminus. Protein kinase CK2 phosphorylates the N-terminus of isoform ECE-1c, increasing its stability and leading to enhanced invasiveness in glioblastoma and colorectal cancer cells, which is believed to be mediated by the amino acid residue Lys-6, a conserved putative ubiquitination site neighboring the CK2-phosphorylated residues Ser-18 and Ser-20. Whether Lys-6 is linked to the acquisition of a cancer stem cell (CSC)-like phenotype and aggressiveness in human non–small cell lung cancer (NSCLC) cells has not been studied.

**Methods:**

In order to establish the role of Lys-6 in the stability of ECE-1c and its involvement in lung cancer aggressiveness, we mutated this residue to a non-ubiquitinable arginine and constitutively expressed the wild-type (ECE-1c^WT^) and mutant (ECE-1c^K6R^) proteins in A549 and H1299 human NSCLC cells by lentiviral transduction. We determined the protein stability of these clones alone or in the presence of the CK2 inhibitor silmitasertib, compared to ECE-1c^WT^ and mock-transduced cells. In addition, the concentration of secreted ET-1 in the growth media was determined by ELISA. Expression of stemness genes were determined by Western blot and RT-qPCR. Chemoresistance to cisplatin was studied by MTS viability assay. Migration and invasion were measured through transwell and Matrigel assays, respectively, and the side-population was determined using flow cytometry.

**Results:**

ECE-1c^K6R^ displayed higher stability in NSCLC cells compared to ECE-1c^WT^-expressing cells, but ET-1 secreted levels showed no difference up to 48 h. Most importantly, ECE-1c^K6R^ promoted expression of the stemness genes c-Myc, Sox-2, Oct-4, CD44 and CD133, which enhance cellular self-renewal capability. Also, the ECE-1c^K6R^-expressing cells showed higher cisplatin chemoresistance, correlating with an augmented side-population abundance due to the increased expression of the ABCG2 efflux pump. Finally, the ECE-1c^K6R^-expressing cells showed enhanced invasiveness, which correlated with the regulated expression of known EMT markers.

**Conclusions:**

Our findings suggest an important role of ECE-1c in lung cancer. ECE-1c is key in a non-canonical ET-1-independent mechanism which triggers a CSC-like phenotype, leading to enhanced lung cancer aggressiveness. Underlying this mechanism, ECE-1c is stabilized upon phosphorylation by CK2, which is upregulated in many cancers. Thus, phospho-ECE-1c may be considered as a novel prognostic biomarker of recurrence, as well as the CK2 inhibitor silmitasertib as a potential therapy for lung cancer patients.

**Supplementary Information:**

The online version contains supplementary material available at 10.1186/s40659-024-00551-9.

## Introduction

Lung cancer is currently the leading cause of death by cancer worldwide [1], rendering this disease a major public health problem. Over 80% of incidence corresponds to non-small cell lung cancer (NSCLC), and the main first-line treatment for advanced stages is cisplatin chemotherapy [2, 3]. Despite this, a considerably high recurrence rate occurs following cisplatin treatment and a poor 5-year survival of 4–17% is observed depending on stage and location [3, 4]. As with many other cancer types, recurrence in lung cancer has been associated to cells with a stemness phenotype, known as cancer stem cells (CSCs) [5–7].

CSCs correspond to a marginal cell population detected in many tumors, which account for 0.1–1% of the total cell mass, responsible for genesis, therapy resistance, metastasis and recurrence [8–10]. A common CSCs trait is the elevated expression of genes related to stemness such as Nanog, Sox-2, Oct-4 and LGR5, as well as CD44 and CD133 surface markers, among others such as ALDH, CD24, CD166 and EpCAM [9, 10]. Lung CSCs have been indeed reported to express BMI-1, Oct-4, CD44 and CD133, as well as many others such as ABCG2, CD90, CD177, CD166 and Nanog [11–13]. Another characteristic of CSCs is the augmented expression of ATP-binding cassette (ABC) transporters, such as P-glycoprotein, ABCC1 and ABCG2, which can pump several types of small molecules to the extracellular space, including chemotherapeutic drugs, thus allowing them to resist chemotherapy and ultimately promoting recurrence in patients, even several years after tumor regression [13–17]. Despite the above advances, the precise molecular mechanisms leading to the acquisition of CSCs attributes are still not fully understood.

The endothelin-1 (ET-1) axis has been involved in lung cancer aggressiveness and suggested as an indicator of poor prognosis [18]. ET-1 expression has been associated to stemness, being detected in CSCs derived from several tumor types, including lung cancer [19]. Moreover, ET-1 silencing decreases proliferation and invasiveness of A549 human lung cancer cells [20]. ET-1 is a small mitogenic peptide extracellularly activated by the endothelin-converting enzyme-1 (ECE-1). This enzyme is expressed in almost all cells as four isoforms, which only differ by their cytoplasmic N-terminus of 52–68 residues, depending of species. Nevertheless, the ECE-1c isoform is the most highly expressed isoform in several cancers [21].

We reported that protein kinase CK2 can phosphorylate the N-terminal end of isoform ECE-1c at Ser-18 and Ser-20, which enhances its stability and, importantly, triggers aggressiveness of colorectal cancer cells [22, 23]. An elevated expression and activity of CK2 has been detected in many types of cancer cells, including lung cancer [24, 25]. CK2 can promote survival through activation of b-catenin at the canonical Wnt pathway [26], which increases the expression of many targets, including survivin and COX-2 [27, 28]. In fact, ET-1 is another b-catenin target whose expression increases in several cancers, including lung cancer, however, ET-1’s canonical mitogenic effects depend on its continuous activation by the enzyme ECE-1 [21]. Thus, we assessed whether ECE-1c can potentially promote aggressiveness of lung cancer cells. Here, we report, in A549 and H1299, human lung cancer cells, a non-canonical ET-1-independent mechanism in which expression of a highly stable ECE-1c protein leads to a CSC-like phenotype and hence aggressiveness features, accounting for the elevated recurrence observed in cisplatin-treated patients.

## Results

### Lysine 6 is related to higher stability of ECE-1c in lung cancer cells

The N-terminal end of ECE-1c from several species shows a conserved lysine at position 6, which is quite close to the Ser-18 and Ser-20 phosphorylated by CK2 [23]. In order to potentially disturb Lys-6 ubiquitination and to assess the role of ECE-1c stability in human NSCLC cells, a point-mutation Lys-to-Arg (i.e. K6R) was performed. Lentiviral vectors harboring either ECE-1c^K6R^, ECE-1c^WT^ or mock (empty vector) were constructed in a bicistronic unit with the fluorescent protein mCherry. These lentiviruses were used to transduce two human NSCLC cell lines, A549 and H1299, whose subsequent three individual clones were then expanded and sorted by flow cytometry based on mCherry fluorescence. Of note, both NSCLC cell lines had significantly augmented endogenous mRNA levels of isoform ECE-1c compared to the other three isoforms (Sup Fig. 1).

**Fig. 1:**
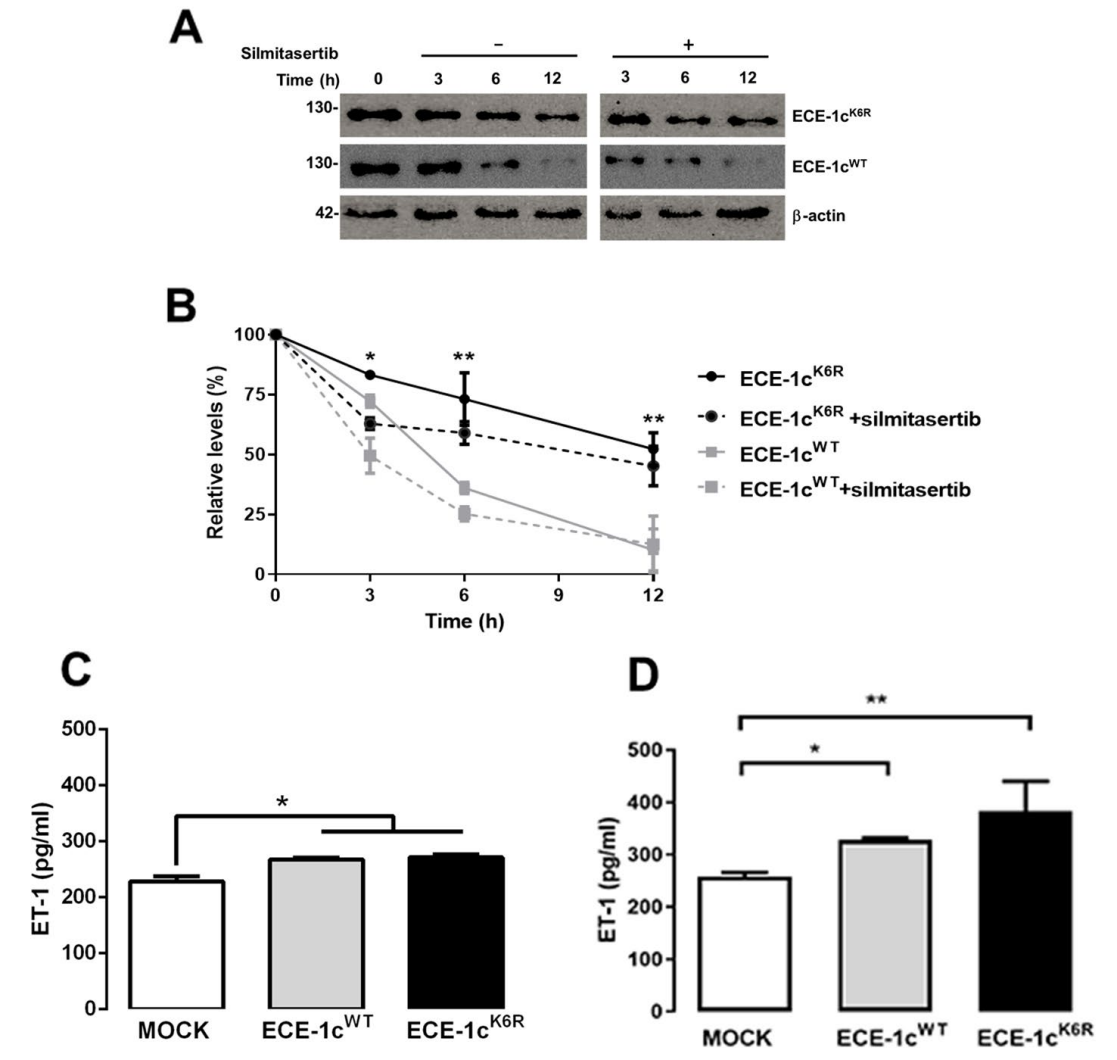
ECE-1c^K6R^ mutant is highly stable in A549 cells. (**A**) ECE-1c^WT^- and ECE-1c^K6R^-expressing cells were treated with cycloheximide (CHX) in the absence or presence of CK2 inhibitor silmitasertib. Protein levels were evaluated by Western blot with an anti-Flag antibody, using β-actin as loading control. Representative blots are shown. (**B**) Relative levels of ECE-1c proteins from three independent experiments were calculated. (**C**,**D**) Clones described in A were grown for 48 h without FBS. ET-1 levels were measured in supernatants after 24 (**C**) and 48 h (**D**) of growth by ELISA, according to manufacturer’s instructions. Values were plotted as mean ± SE from at least three independent experiments performed in triplicate. *P ≤ 0.05, **P ≤ 0.01

Mock, ECE-1c^WT^- and ECE-1c^K6R^-expressing A549 clones were grown in the presence of cycloheximide (CHX) to prevent protein synthesis, but also in the absence or presence of a specific CK2 inhibitor, silmitasertib (also known as CX-4945), to induce protein degradation, as reported elsewhere [22]. ECE-1c protein levels were detected by Western blot up to 12 h, whose results showed that ECE-1c^K6R^ is highly stable in comparison to ECE-1c^WT^ in A549 cells (Fig. 1A). In addition, ECE-1c^K6R^ protein levels were also higher compared to ECE-1c^WT^ in H1299 lung cancer cells (Sup Fig. 2). Moreover, although ECE-1c^K6R^ levels were affected in a lower degree by CK2 inhibition, ECE-1c^WT^ protein levels were significantly diminished as compared to ECE-1c^K6R^ in the absence and, even more so, in the presence of silmitasertib (Fig. 1B).

In order to evaluate whether this marked difference in protein stability impacts on ET-1 production, secreted levels of this peptide were measured in the culture media of A549 cells at different times. Secreted ET-1 levels from both ECE-1c^WT^- and ECE-1c^K6R^-expressing cells were significantly higher as compared to mock cells at 12 h (not shown), 24 h (Fig. 1C) and 48 h (Fig. 1D) but, interestingly, ET-1 levels displayed no significant difference between ECE-1c^WT^- and ECE-1c^K6R^-expressing cells, showing only a non-significant increase in ECE-1c^K6R^-expressing cells at 48 h. Altogether, these results demonstrate a key role of Lys-6 in protein stability of ECE-1c in NSCLC cells, although it may not affect the production of ET-1, which was indistinguishable between both ECE-1c-expressing cells.

### Stable ECE-1c^K6R^ expression promotes stemness traits

**Fig. 2:**
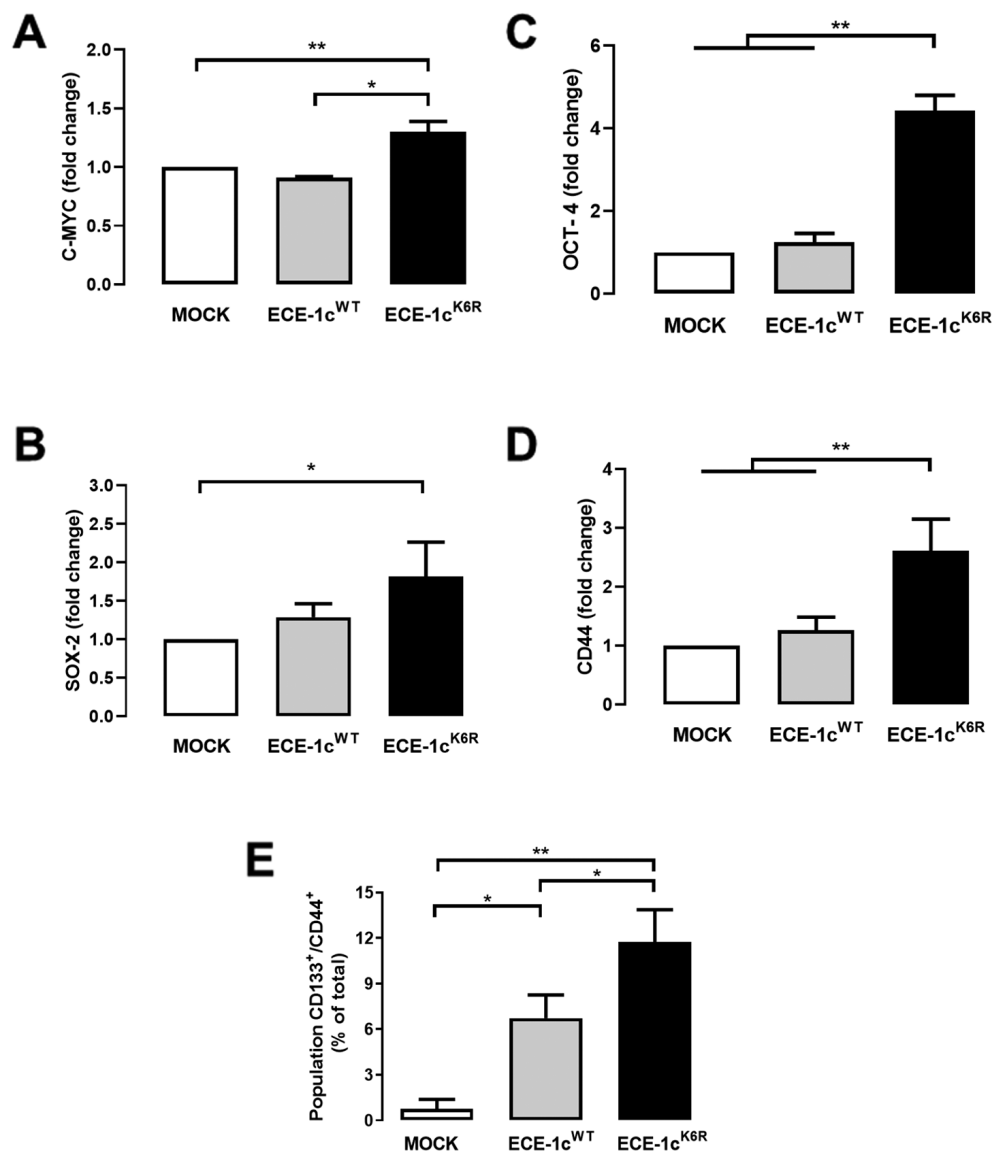
ECE-1c^K6R^ promotes expression of stemness genes. Mock, ECE-1c^WT^- and ECE-1c^K6R^-expressing A549 cells were grown under normal conditions for 24 h. Then, mRNA levels of stemness genes c-Myc (**A**), Sox-2 (**B**), Oct-4 (**C**) and CD44 (**D**) were quantified by RT-qPCR. (**E**) Cells grown as above were evaluated for CD133^+^/CD44^+^ population using flow cytometry and FLOWJO V.10 software. Values were plotted as mean ± SE from at least three independent experiments performed in triplicate. *P ≤ 0.05, **P ≤ 0.01

ECE-1c^WT^ and highly stable ECE-1c^K6R^ proteins were evaluated in their capacity to trigger occurrence of stemness traits. As observed, ECE-1c^K6R^ expression in A549 cells promoted significantly higher mRNA levels of several stemness genes, namely c-Myc, Sox-2 and Oct-4 (Fig. 2A-C). Likewise, ECE-1c^K6R^ expression in H1299 cells induced significantly higher mRNA levels of the stemness gene Oct-4 (Sup Fig. 2A). However, other two known stemness genes, BMI-1 and Stat-3, did not show any significant difference between mock, ECE-1c^WT^- and ECE-1c^K6R^-expressing A549 cells (Sup Fig. 3A, B). According to increased CD44 mRNA levels found in A549 cells (Fig. 2D), a significant higher double-positive CD44^+^/CD133^+^ population was measured by flow cytometry in ECE-1c^K6R^-expressing cells, as compared to mock and ECE-1c^WT^-expressing cells (Fig. 2E).

**Fig. 3:**
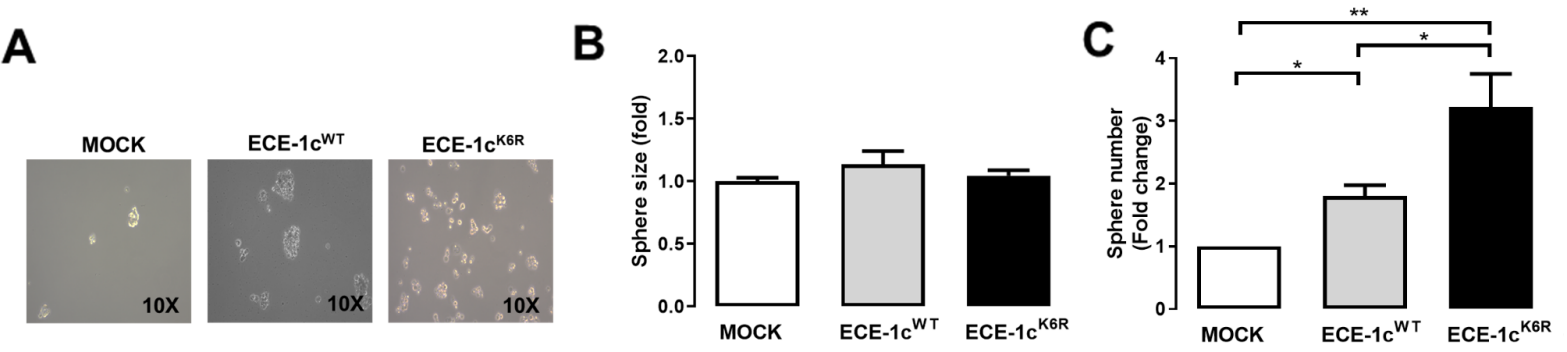
Enhanced sphere formation in ECE-1c^K6R^-expressing cells. Mock, ECE-1c^WT^- and ECE-1c^K6R^-expressing A549 cells were grown for 7 days under spheroidogenic conditions. (**A**) Representative images are shown at 10X magnification. (**B**) Spheroid size was quantified from a triplicate analysis by using the Micrometrics SE Premium 4 software. (**C**) Spheroids were quantified from a triplicate analysis. Values were plotted as mean ± SE from at least three independent experiments performed in triplicate. *P ≤ 0.05, **P ≤ 0.01

We then studied the effect on cellular self-renewal in each A549 clone through a sphere formation assay (Fig. 3A). The results showed that sphere size among mock, ECE-1c^K6R^- and ECE-1c^WT^-expressing cells was very similar (Fig. 3B), however, a significant three-fold increase in the number of spheres was formed in ECE-1c^K6R^-expressing cells (Fig. 3C). Taken together, these results suggest that expression of the highly stable ECE-1c^K6R^ enhances CSC traits in NSCLC cells, which display elevated levels of stemness and surface marker genes, as well as the ability to self-renew under non-adherent conditions.

### Chemoresistance is enhanced in ECE-1c^K6R^-expressing cells

**Fig. 4:**
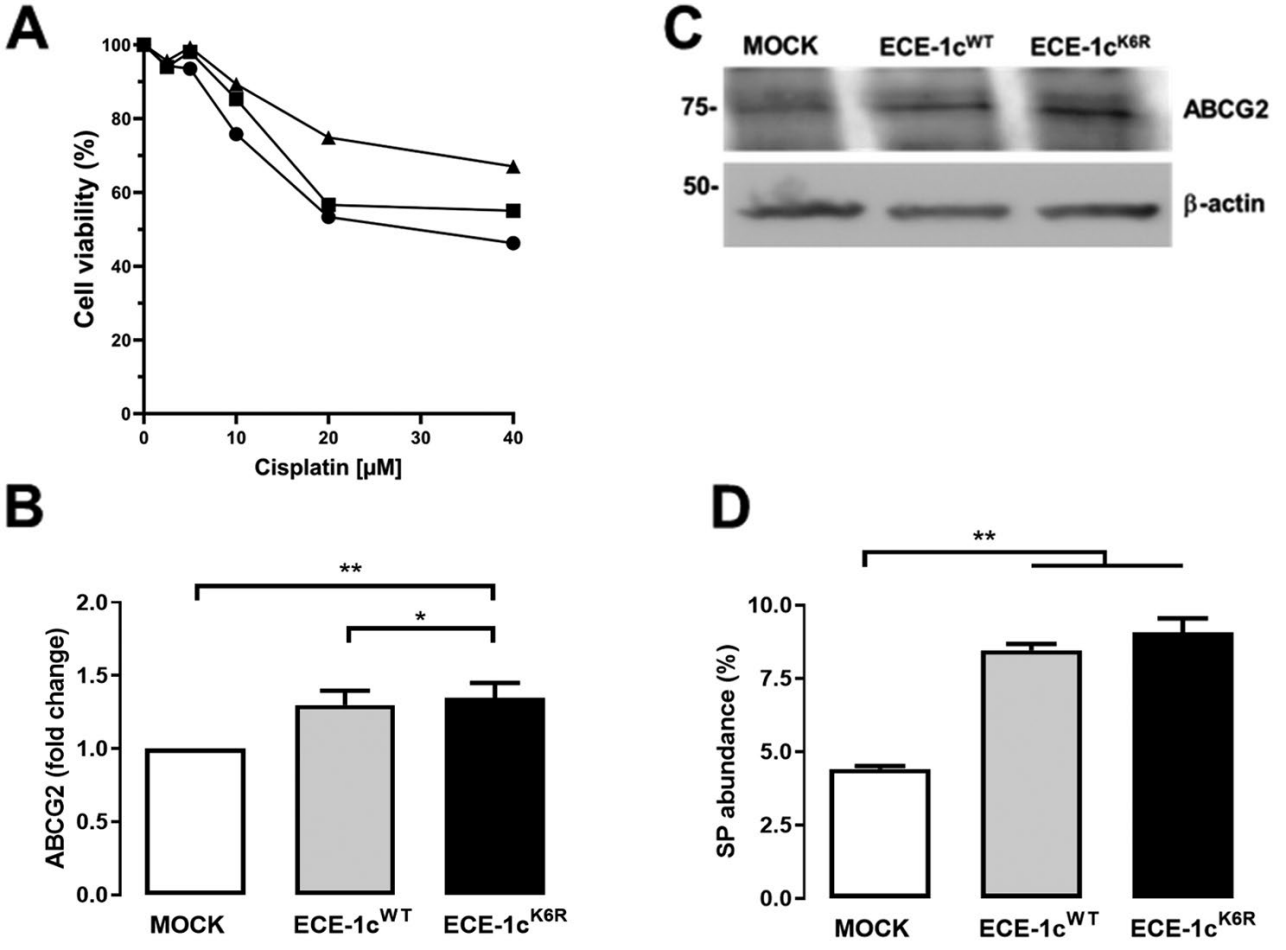
ECE-1c^K6R^ expression leads to enhanced drug resistance. (**A**) Mock (circle), ECE-1c^WT^ (square) and ECE-1c^K6R^ (triangle) expressing A549 cells were grown in presence of 0, 2.5, 5, 10, 20 and 40 µM cisplatin for 48 h, and viability measured using the MTS assay. Mean values were plotted from two independent experiments performed in triplicate. (**B**,**C**) Cells grown under normal conditions for 24 h were analyzed for mRNA (**B**) and protein (**C**) levels of ABCG2 by RT-qPCR and Western blot, respectively. (**D**) Cells were grown under normal conditions for 24 h, treated in the presence or absence of verapamil, and then incubated with DyeCycle Violet (DCV). Side population (SP) abundance was determined by flow cytometry. Values were plotted as mean ± SE from at least three independent experiments performed in triplicate. *P ≤ 0.05, **P ≤ 0.01

Given the role of CSCs in the development and aggressiveness of several cancers, including lung cancer [16, 17], resistance to the antineoplastic drug cisplatin was evaluated in the three A549 clones. A dose-response analysis showed an augmented chemoresistance of cells expressing the highly stable ECE-1c^K6R^ protein as compared to ECE-1c^WT^-expressing and mock cells, specially at concentrations over 10 µM cisplatin (Fig. 4A). In comparison to A549, H1299 cells were even more sensitive to cisplatin (Sup Fig. 4A), by which a concentration of 10 µM was used to evaluate resistance to the antineoplastic. This analysis showed that both mock and ECE-1c^WT^-expressing cells displayed the same viability, while ECE-1c^K6R^-expressing cells displayed over a 50% higher viability compared to mock cells (Sup Fig. 4B). Altogether, these results suggest that the expression of a highly stable ECE-1c^K6R^ in NSCLC cells leads to significantly enhanced chemoresistance.

Augmented ABC pump gene expression has been associated with chemoresistance, attributable to the efflux of drugs from cells [14, 15]. Indeed, both ECE-1c^WT^- and ECE-1c^K6R^-expressing A549 cells showed a significant 25% increase in mRNA (Fig. 4B) and protein (Fig. 4C) levels of ABCG2 in comparison to mock cells after 24 h of growth. According to this, similar increases in mRNA (Sup Fig. 2B) and protein (Sup Fig. 2C) levels were observed in H1299 cells expressing the highly stable ECE-1c^K6R^ enzyme. In addition, we also measured in A549 cells the relative mRNA levels of another known pump, ABCC1, but these were statistically similar under all conditions (Sup Fig. 3C). Finally, in order to correlate the augmented ABCG2 mRNA and protein levels with a functional trait in A549 cells, the side population (SP) abundance was determined by flow cytometry with the dye DCV in the absence or presence of the pump inhibitor verapamil. As shown in Fig. 4D, ECE-1c^WT^- and ECE-1c^K6R^-expressing cells displayed a similar SP fraction of DCV-negative cells (with a slightly higher tendency in ECE-1c^K6R^ cells), but both clones showed a significant two-fold higher increase in SP compared to mock cells. These results indicate that an augmented ECE-1c protein level significantly promotes cisplatin-resistance presumably triggered by up-regulated expression of the ABCG2 pump in NSCLC cells.

### ECE-1c^K6R^ expression leads to metastasis-associated traits

**Fig. 5:**
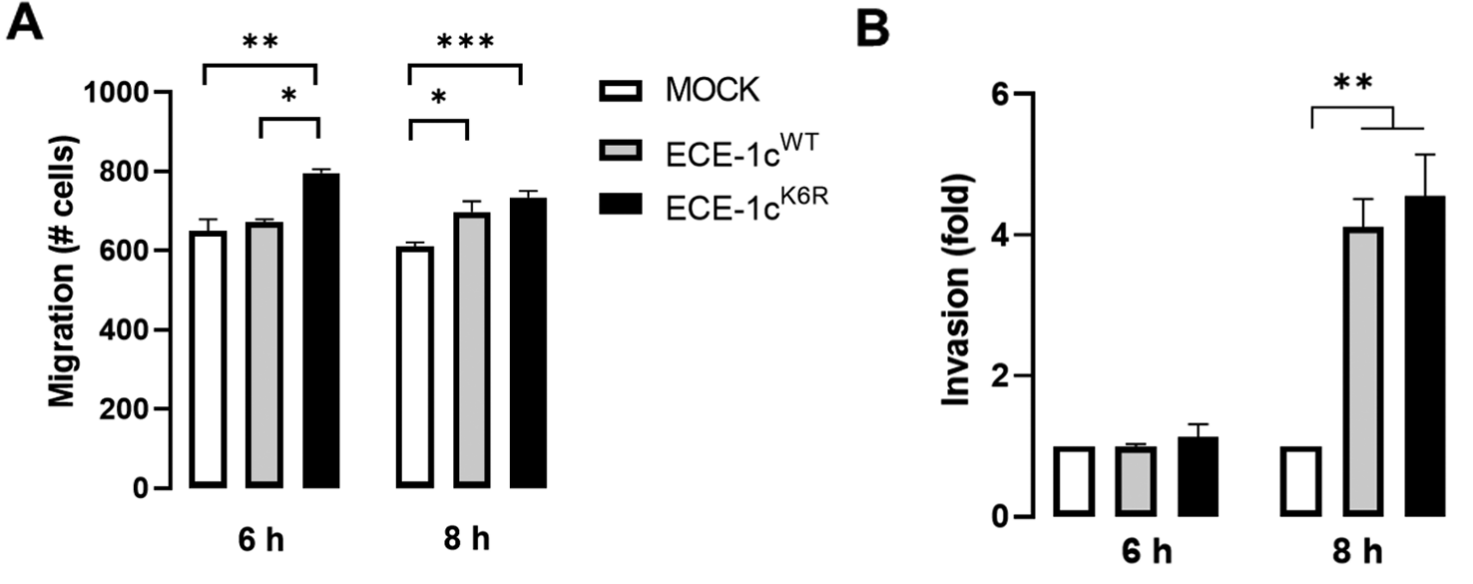
Migration and invasion are enhanced in ECE-1c^K6R^-expressing cells. (**A**) Migration capacity of mock, ECE-1c^WT^- and ECE-1c^K6R^-expressing A549 cells was evaluated at 6 and 8 h of incubation in a transwell chamber. (**B**) Invasion capacity of cells as in A was evaluated at 6 and 8 h of incubation in a Matrigel assay. Cells were counted and plotted for each cell clone. Values were plotted as mean ± SE from at least three independent experiments performed in triplicate. **P ≤ 0.01

**Fig. 6:**
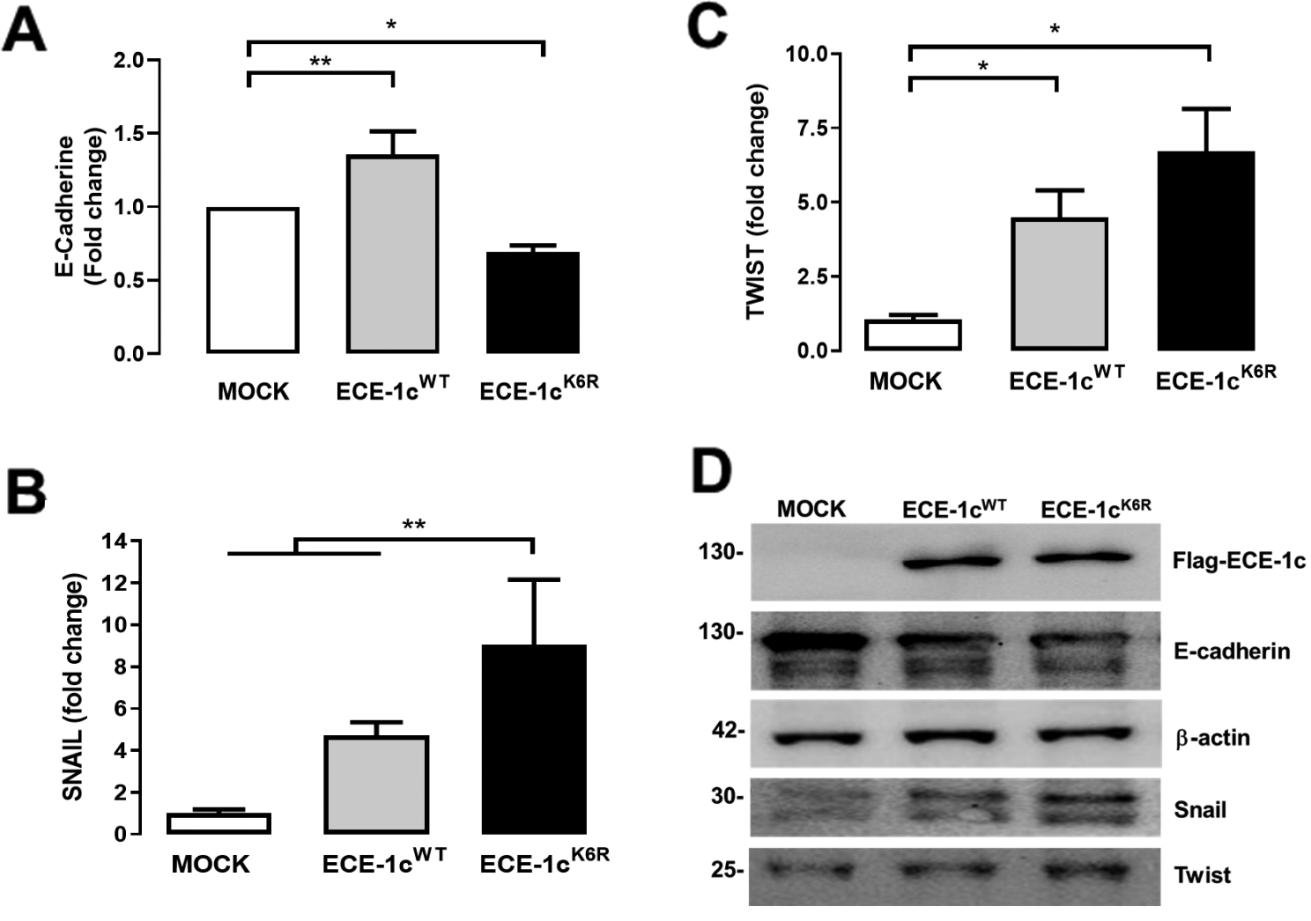
ECE-1c^K6R^ modulates expression of EMT-related markers. Mock, ECE-1c^WT^- and ECE-1c^K6R^-expressing A549 cells were grown under normal conditions for 24 h. Transcript levels of E-cadherin (**A**), Snail (**B**) and Twist (**C**) were quantified by RT-qPCR. (**D**) Protein levels of E-cadherin, Snail and Twist from cells grown as in A were detected by Western blot with specific antibodies. ECE-1c was detected with an anti-Flag antibody. Representative blots are shown. Values were plotted as mean ± SE from at least three independent experiments performed in triplicate. *P ≤ 0.05, **P ≤ 0.01

CSCs are known to display migration and invasion capabilities, which are related to tumor infiltration and metastasis [8]. Thus, we evaluated the effect of highly stable ECE-1c^K6R^ expression on the migration potential of our NSCLC clones using a transwell assay. The results showed a significantly elevated migration capacity of ECE-1c^K6R^-expressing cells in comparison to mock and ECE-1c^WT^-expressing cells at 6 and 8 h, in both A549 (Fig. 5A) and H1299 (Sup Fig. 5A) cells. On the other hand, a Matrigel-coated invasion assay with our A549 cells showed no difference in invasiveness of the three clones at 6 h. However, an over four-fold higher invasiveness was displayed by both ECE-1c^WT^- and ECE-1c^K6R^-expressing cells at 8 h (Fig. 5B). In contrast to A549 cells, a significant increase in invasiveness was observed in ECE-1c^WT^- and ECE-1c^K6R^-expressing H1299 cells compared to mock cells at 6 and 8 h, although with no significant differences among the two ECE-1c-expressing clones (Sup Fig. 5B). Importantly, this aggressiveness trait was accompanied by changes in the expression of known EMT markers following ECE-1c^K6R^-expression in A549 cells. E-cadherin mRNA (Fig. 6A) and protein (Fig. 6D) levels decreased significantly, while mRNA levels of Snail (Fig. 6B), Twist (Fig. 6C) and N-cadherin (Sup Fig. 3D), as well as protein levels of Snail and Twist (Fig. 6D) augmented significantly. Altogether, these results show that a highly stable ECE-1c promotes an enhanced invasiveness in NSCLC cells presumably due to EMT activation.

## Discussion

Most proteins are polyubiquitinated in lysine to be further degraded by the proteasome [29, 30]. In fact, angiotensin-converting enzyme-2 (ACE-2), a member of the same metalloproteinase family, is ubiquitinated and degraded by the proteasome in the lung epithelium [31]. In this work we show that the exchange of Lys-6 for a non-ubiquitinable residue, arginine, renders ECE-1c highly resistant to proteasomal degradation in NSCLC cells. Lys-6 is proximal to the reported Ser-18 and Ser-20 residues phosphorylated by CK2 [23]. Of note, ECE-1c stability was CK2 phosphorylation-dependent, since pharmacological inhibition with silmitasertib decreased ECE-1c^WT^ protein levels at a higher rate than ECE-1c^K6R^.

Nevertheless, the ECE-1c^K6R^ protein was still sensitive to silmitasertib, which had already been observed in glioblastoma and colorectal cancer cells [32, 33]. This suggests that CK2 may be playing a role in ECE-1c degradation through a parallel proteolytic pathway. In fact, silmitasertib leads to mTORC1 inhibition and autophagy traits in cancer cells [34–36]. Additionally, this inhibitor promotes methuosis-like death associated to massive macropinocytosis in colorectal cancer cells [36]. Regarding this, macropinocytosis-internalized proteins undergo degradation upon fusion with lysosomes [37]. So, ECE-1c may be internalized and degraded through this endosome-lysosome pathway. Therefore, it is plausible that at least a pool of ECE-1c may be still subject to degradation via those parallel proteolytic pathways. Moreover, it has been recently reported that a phosphomimetic ECE-1c mutant displayed significant resistance to ubiquitination [38]. Likewise, the proteasomal inhibitor, MG-132, did block silmitasertib-triggered degradation in colorectal cancer cells [22]. Thus, CK2-phosphorylation of ECE-1c seems to protect it mainly from proteasomal degradation.

**Fig. 7:**
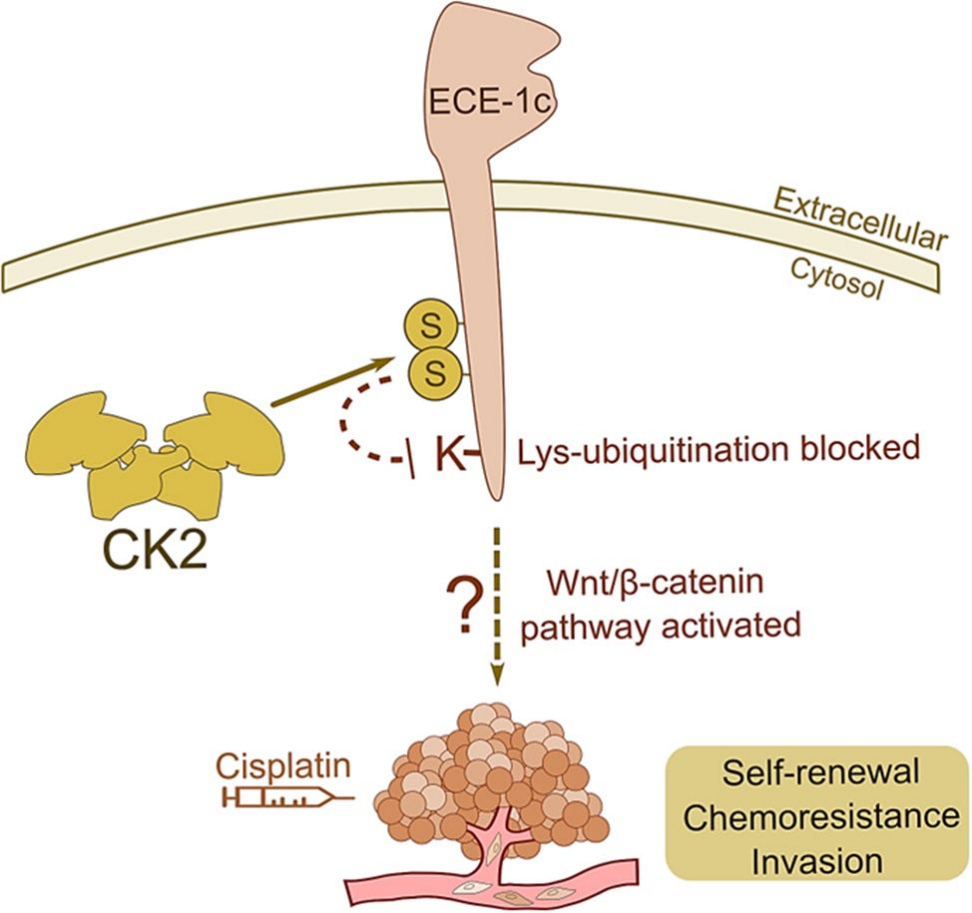
A model for post-translational regulation of ECE-1c’s stability and its role in aggressiveness of NSCLC cells. The findings reported here support a mechanism of aggressiveness operating in ECE-1c-expressing A549 and H1299 cells which is independent of the production of ET-1. This may function through bypass to other pathways related with aggressiveness, such as the Wnt/b-catenin signaling pathway, which frequently involve the upregulation of aggressiveness-related genes, including Akt, COX-2, FAK, matrix-metalloproteases, etc [43, 44, 48, 49]. Underlying this mechanism, stabilization of ECE-1c is crucial, putatively through CK2-dependent phosphorylation

Literature shows that CK2 can regulate the stability of many other proteins, for example, OTUB1 phosphorylation promotes its nuclear deubiquitinase activity [39]. Also, CK2 phosphorylation of c-Myc prevents its proteasomal degradation, enhancing the transcription of genes involved in several cancer hallmarks [40]. Moreover, CK2 phosphorylation of HSP90 promotes expression of P-glycoprotein in colorectal cancer cells by cytoplasmic accumulation of PXR [41]. Nevertheless, although the underlying molecular mechanism of ECE-1c gain of stability remains unclear, we have shown here that Lys-6 has a key role in its proteasomal degradation, which may be eventually blocked by CK2-mediated phosphorylation (Fig. 7).

The improved stability of ECE-1c by blockage of ubiquitination at Lys-6 also dramatically promoted in vitro stem-like cells traits in human A549 and H1299 lung cancer cells, which is a novel finding of this work. The augmented number of spheres was consistent with an elevated expression of stemness-related genes such as Sox-2, Oct-4, CD44 and CD133, the latter correlating with that already reported for NSCLC elsewhere [13]. Although BMI has also been reported as a potential NSCLC stem cell marker, its mRNA levels were quite similar in the three independent lung cancer cell clones, probably indicating that its known function in proliferation and senescence [13] was dispensable in ECE-1c-expressing cells. On the other hand, c-Myc, whose levels are augmented in NSCLC cells, is not particularly signed as a stemness gene. However, it was significantly elevated in ECE-1c^K6R^-expressing cells, which may be related to a Sox-2-dependent regulation of its expression [42].

An enhanced expression of the above stemness genes strongly suggests a link between the ET-1 axis and CSCs. In fact, augmented transcript levels of the ET-1 peptide and ECE-1 have been detected in NSCLC samples compared to their adjacent normal tissue [20]. Likewise, overall survival and disease-free interval curves of lung cancer patients in relation to ET-1 mRNA levels suggest that this would be a poor prognosis biomarker [18]. However, our results showed that the higher levels of secreted ET-1 are not responsible for the significantly higher aggressiveness of cells expressing the highly stable ECE-1c^K6R^, since ET-1 levels were quite similar to those found in the growth medium of ECE-1c^WT^-expressing cells.

We have shown here that the expression of a highly stable ECE-1c^K6R^ protein promotes a significant higher chemoresistance to cisplatin when compared to mock cancer cells, which indeed have augmented endogenous mRNA ECE-1c levels (Sup Fig. 1). In this respect, our results showed that although ABCC1 and ABCG2 were evaluated, only ABCG2 increased significantly, in agreement with that reported elsewhere [13], and also functionally participated in pumping out the drug, accounting for the higher chemoresistance in ECE-1c-expressing cells. Taken together, based on the elevated expression detected for both stemness and ABC-pump genes in ECE-1c^K6R^-expressing NSCLC cells, a putative mechanism of chemoresistance may involve activation of bypass signaling pathways [43, 44], which is consistent with the fact that an augmented expression of this enzyme is related to poor prognosis in NSCLC [18].

The enhanced invasiveness observed in ECE-1c^K6R^-expressing cells may be a consequence of the differences in expression of EMT markers, such as E-cadherin, Snail and Twist. In fact, high levels of Twist and CD133 as well as low levels of E-cadherin, together with high nuclear b-catenin and low Sox15, have been proposed as diagnostic markers in lung cancer [13]. Similarly, ECE-1c levels has been correlated with invasiveness, EMT and high ET-1 levels in ovarian cancer cells [45], as well as exogenous ET-1-induced migration and MMP expression in GBM cells [46]. This suggests that the effect of ECE-1c in lung cancer cells may also be dependent on ET-1 production. However, as discussed above, secreted ET-1 levels in ECE-1c^K6R^ growth medium were quite similar to those found in ECE-1c^WT^-expressing cells at both 24 and 48 h. Therefore, ET-1 may be not responsible for the significantly higher invasiveness of cells expressing stable ECE-1c^K6R^. This correlates with that observed in prostate cancer cells in which exogenous ET-1 did not rescue the low invasiveness following ECE-1c silencing [47].

Therefore, a non-canonical ET-1-independent mechanism may account for the higher aggressiveness observed in tumors that express elevated levels of the ECE-1c protein [21]. Despite the above experimental evidence, an in silico analysis of public databases does not show the occurrence of any mutation at Lys-6 of ECE-1c which otherwise may allow to extrapolate our findings to lung cancer patients. However, the findings presented here and in previous reports [32, 33] provides proof-of concept as to the role of ECE-1c in cancer progression. Furthermore, the fact that the activity and protein levels of CK2 are found elevated in the majority of the cancers [24, 25] suggests a putative post-translational mechanism of aggressiveness operating at least in glioblastoma, colorectal and, as shown in this report, lung cancer cells.

## Materials and methods

### Cell culture and lentiviral cloning

A549/CCL-185 and H1299/CRL-5803 human non-small cell lung cancer cells (ATCC, Rockville, MD, USA) were grown at 37 °C and 5% CO_2_ in RPMI-1640 medium (Life Technologies, NY, USA) containing 10% FBS (Cytiva, Pasching, Austria) and 100 units/mL penicillin/streptomycin (ThermoFisher Scientific, MA, USA). Once a year, one N_2_ aliquot was thawed, expanded and stored again at -80 °C. For experiments, one − 80 °C aliquot was thawed and grown in normal medium. All experiments were performed within one year and cells were discarded after a maximum of 15 passages. Mycoplasma contamination was tested monthly with the EZ-PCR Mycoplasma Test kit (Biological Industries, Beit Haemek, Israel). For obtention of stable cell clones overexpressing Myc/Flag-tagged (C-terminal end) ECE-1c forms, a pLVX-IRES-mCherry bicistronic lentiviral vector (Takara Bio, CA, USA) was used. Lentiviral vector production was developed using the Lenti-X 293T cell line (Takara Bio, CA, USA) by transfection of a second-generation lentiviral system. Cells were cultured with the lentiviral particles encoding each ECE-1c form, expanded for one week, trypsinized and sorted using FACSAria Fusion equipment (Becton Dickinson, NJ, USA). Gating was performed on the brightest mCherry cells, which were collected and subsequently expanded.

### Protein stability

Cells (1 × 10^6^ per well) were seeded into 12-well plates and cultured overnight at 37 °C and 5% CO_2_ in RPMI-1640 medium supplemented with 10% FBS and then incubated with 20 mg/mL cycloheximide (Sigma-Aldrich, MO, USA) in the absence or presence of 25 µM silmitasertib (Apexbio, TX, USA) or vehicle only (DMSO) for 12 h. Cells were harvested after treatment, lysed and 25 µg of total protein were analyzed by Western blot with an anti-Flag antibody.

### Western blot

Proteins were separated by SDS-PAGE and transferred to nitrocellulose membranes. Membranes were blocked with 5% BSA/TBST and then incubated at 4 °C overnight with primary antibodies diluted in blocking buffer. The primary antibodies used were: Flag-tag (Cell Signaling Technology, MA, USA), Snail (Cell Signaling Technology, MA, USA), Twist (Santa Cruz Biotechnology, TX, USA), E-cadherin (Santa Cruz Biotechnology, TX, USA) and β-actin (Cell Signaling Technology, MA, USA). Blots were then washed three times for 10 min at room temperature with TBST, incubated for 1 h at room temperature with secondary HRP-conjugated antibodies (Santa Cruz Biotechnology, TX, USA) diluted in TBST, washed again and revealed in a Chemidoc instrument (Bio-Rad, CA, USA) using the Clarity Western ECL Substrate (Bio-Rad, CA, USA). B&W images were color-inverted and processed for densitometry analysis using the ImageJ software.

### ET-1 ELISA

Mock, ECE-1c^WT^- and ECE-1c^K6R^-expressing cells (1 × 10^6^ per well) were seeded into 100 mm plates and cultured for 48 h at 37 °C and 5% CO_2_ in RPMI-1640 medium supplemented with 10% FBS. ET-1 levels (pg/mL) were quantified in culture media by using an ET-1 Human ELISA Kit (ThermoFisher Scientific, MA, USA), according to the manufacturer’s instructions, and normalized to 1 mg/mL of total protein.

### Flow cytometry and Side Population assay

Cells (1 × 10^5^ per well) were incubated with 0.25 µg 7-AAD (BioLegend, CA, USA) as a viability marker and then with anti-CD133/APC and anti-CD44/BV-421 antibodies (BioLegend, CA, USA) diluted in 200 µL PBS/2% FBS for 30 min. Unlabeled cells, APC mouse IgG1ƙ and BV-421 mouse IgG1ƙ isotypes (BioLegend, CA, USA) were used as controls. For Side Population assay, cells were treated with 200 µM verapamil (Sigma-Aldrich, MO, USA), incubated for 30 min with Dye Cycle Violet (ThermoFisher Scientific, MA, USA), washed and prepared for analysis in a Becton-Dickinson LSR Fortessa X-20 flow cytometer by using the FACSDIVA 8.02 software (Becton Dickinson, NJ, USA).

### RT-qPCR

Total RNA was extracted with Trizol (ThermoFisher Scientific, MA, USA) and quantified in a NanoDrop instrument. Reverse transcription was performed on 1 µg RNA with MMLV-RT (ThermoFisher Scientific, MA, USA) following manufacturer instructions. qPCR was performed using the ΔΔCt method, with either GAPDH or HPRT1 as normalizer genes, 250 nM of each primer (IDT, IA, USA) using the 5x HOT FIREPol EvaGreen qPCR Mix Plus (Solis BioDyne, Tartu, Estonia) following the manufacturer’s instructions. The sequence of each forward and reverse primer is detailed in Table 1.

**Table 1:**
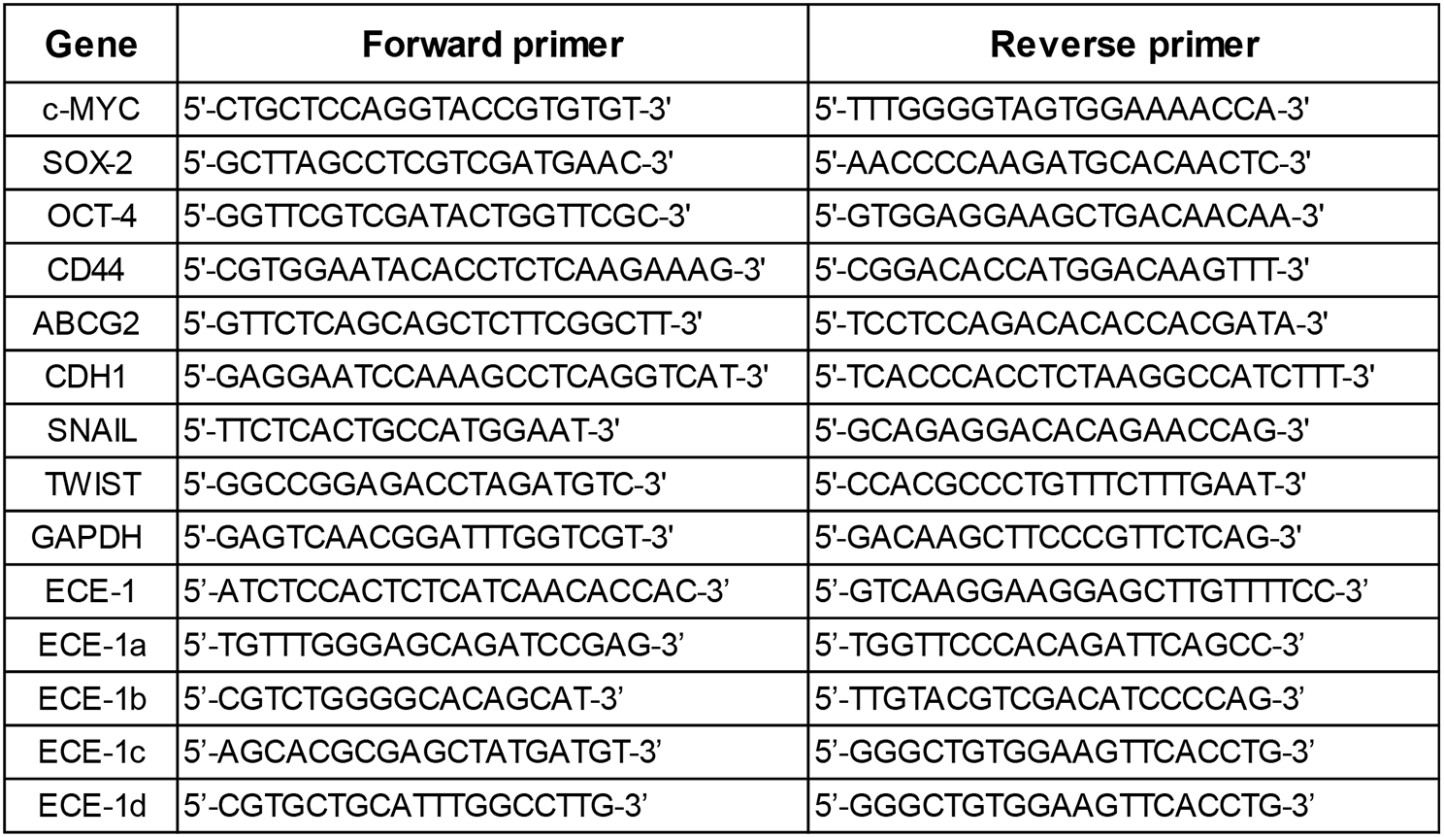
Sequence of primers used in this work

### Spheroid formation

Cells (5 × 10^4^ per well) were seeded into ultra-low attachment 6-well plates (Corning, NY, USA) containing 2% UltraPure Agarose (ThermoFisher Scientific, MA, USA). Cells were cultured at 37 °C, 5% CO_2_ for 7 days in mammary epithelial cell growth media (MEGM) supplemented with 20 ng/mL EGF/0.5 g/mL hydrocortisone/5 µg/mL insulin (Lonza, Basel, Switzerland), and 25 ng/mL b-FGF (ThermoFisher Scientific, MA, USA). Spheres > 100 μm in diameter were counted and measured. Quantification was performed with the Micrometrics SE Premium 4 software (Accu-Scope, NY, USA).

### Chemoresistance

Cells (1 × 10^4^ per well) were seeded in 96-well plates in quadruplicate (Corning, NY, USA). After 24 h, cisplatin or vehicle (DMSO) were added. Proliferation was measured after 48 h using the CellTiter 96 AQueous Non-Radioactive Cell Proliferation Assay (Promega, WI, USA) according to the manufacturer’s instructions. Absorbance was read at 490 nm on a NanoQuant Infinite M200 spectrophotometer (Tecan, Männedorf, Switzerland).

### Migration and invasion

Cells were plated on the top side of a polycarbonate Transwell chamber (Corning, NY, USA) for migration assay or in a matrigel-coated Transwell chamber (Corning, NY, USA) for invasion assay. Cells (7.5 × 10^4^ per chamber) were seeded in serum-free RPMI-1640 and RPMI-1640/10% FBS was deposited in the lower chamber as chemoattractant medium. Cells were incubated at 37 °C for different times. Cells in the top chamber were carefully removed with cotton swabs and migrated/invaded cells were fixed in 3.7% p-formaldehyde/PBS and stained with 1% crystal violet/PBS during 10 min. Cells were counted under the 10X objective in 5 different fields of the insert underside. The mean number of cells was normalized to 1 using the mock condition and then plotted.

### Statistical analysis

Statistical analysis and plotting were done with GraphPad Prism 6.01 software (San Diego, CA, USA). Statistical analysis was performed on raw data using ANOVA and Tukey as post-test. A P-value ≤ 0.05 was considered significant.

## Electronic supplementary material

Below is the link to the electronic supplementary material.

Supplementary Figure 1. Increased mRNA levels of ECE-1c isoform in lung cancer cells. (A) Relative total mRNA levels of ECE-1 were determined by RT-qPCR in A549 and H1299 cell lines, using the HPRT1 gene as normalizer. (B,C) Relative mRNA levels of ECE-1 isoforms were determined in A549 (B) and H1299 (C) cells as in A. Mean values were plotted from two independent experiments performed in triplicate

Supplementary Figure 2. ECE-1c^K6R^ promotes expression of stemness genes in H1299 lung cancer cells. (A,B) Mock, ECE-1c^WT^- and ECE-1c^K6R^-expressing cells were grown under normal conditions for 24 h. Then, mRNA levels of stemness genes Oct-4 (A) and ABCG2 (B) were quantified by RT-qPCR. Mean values were plotted from two independent experiments performed in triplicate. (C) Mock, ECE-1c^WT^- and ECE-1c^K6R^-expressing cells were grown as in A. Levels of Flag-ECE-1c proteins and ABCG2 were detected by Western blot with anti-Flag and anti-ABCG2 specific antibodies, using β-actin as loading control. Representative blots are shown from two independent experiments.

Supplementary Figure 3. Transcript levels of BMI-1, Stat-3, ABCC1 and N-cadherin in ECE-1c-expressing A549 lung cancer cells. Mock, ECE-1c^WT^- and ECE-1c^K6R^-expressing A549 human lung cancer cells were grown under normal conditions for 24 h. Then, mRNA levels of BMI-1 (A), Stat-3 (B), ABCC1 (C) and N-cadherin (D) were quantified by RT-qPCR. Values were plotted as mean ± SE from at least three independent experiments performed in triplicate. *P ≤ 0.05.

Supplementary Figure 4. ECE-1c^K6R^ expression leads to enhanced drug resistance in H1299 lung cancer cells. (A) Dose-response analysis performed by incubating non-transduced cells with 0, 2.5, 5, 10, 20 and 40 μM cisplatin for 48 h. Viability was measured by the MTS assay. DC_50_ was calculated in 14.4 μM cisplatin. (B) Mock, ECE-1c^WT^- and ECE-1c^K6R^-expressing cells were grown under normal conditions in presence of 10 μM cisplatin for 48 h, and viability was measured as in A. Mean values were plotted from two independent experiments performed in triplicate.

Supplementary Figure 5. Migration and invasion are enhanced in ECE-1c^K6R^-expressing H1299 lung cancer cells. (A) Migration capacity of mock, ECE-1c^WT^- and ECE-1c^K6R^-expressing cells was evaluated in transwell chambers for 6 and 8 h. (B) Invasion capacity of cells as in A was evaluated by a Matrigel assay at 6 and 8 h. Cells were counted for each cell clone and values were plotted as mean ± SE from three independent experiments performed in triplicate. *P ≤ 0.05, **P ≤ 0.01.

## Data Availability

The datasets used and/or analyzed during the current study are available from the corresponding author on reasonable request.
