# Supplementary figures and images for: Cisplatin-resistance and aggressiveness are enhanced by a highly stable endothelin-converting enzyme-1c in lung cancer cells
Source: Biol Res. 2024 Oct 24;57:74. doi: 10.1186/s40659-024-00551-9 (PMC11515556; doi:10.1186/s40659-024-00551-9)

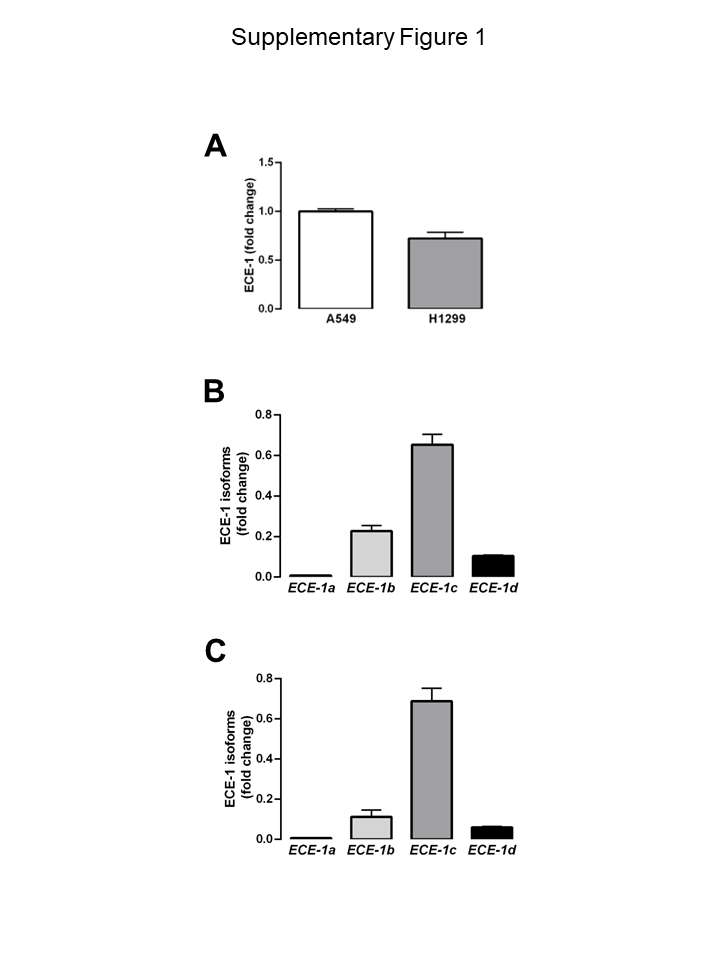

Supplement: Supplementary file 1 — Supplementary Figure 1. Increased mRNA levels of ECE-1c isoform in lung cancer cells. (A) Relative total mRNA levels of ECE-1 were determined by RT-qPCR in A549 and H1299 cell lines, using the HPRT1 gene as normalizer. (B,C) Relative mRNA levels of ECE-1 isoforms were determined in A549 (B) and H1299 (C) cells as in A. Mean values were plotted from two independent experiments performed in triplicate [file 40659_2024_551_MOESM1_ESM.tif]

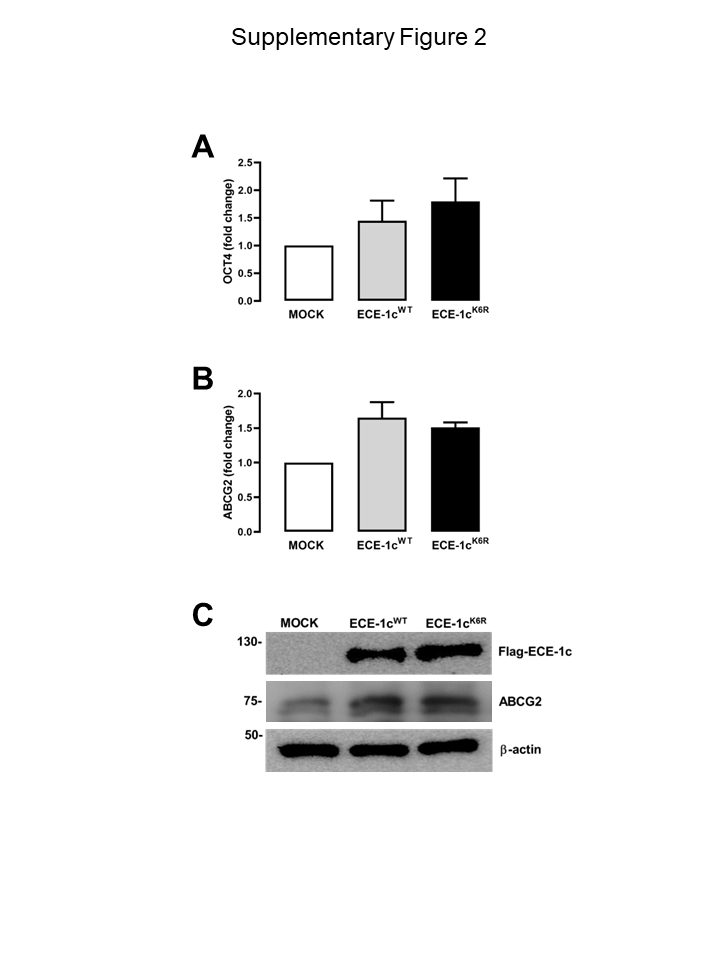

Supplement: Supplementary file 2 — Supplementary Figure 2. ECE-1cK6R promotes expression of stemness genes in H1299 lung cancer cells. (A,B) Mock, ECE-1cWT- and ECE-1cK6R-expressing cells were grown under normal conditions for 24 h. Then, mRNA levels of stemness genes Oct-4 (A) and ABCG2 (B) were quantified by RT-qPCR. Mean values were plotted from two independent experiments performed in triplicate. (C) Mock, ECE-1cWT- and ECE-1cK6R-expressing cells were grown as in A. Levels of Flag-ECE-1c proteins and ABCG2 were detected by Western blot with anti-Flag and anti-ABCG2 specific antibodies, using β-actin as loading control. Representative blots are shown from two independent experiments. [file 40659_2024_551_MOESM2_ESM.tif]

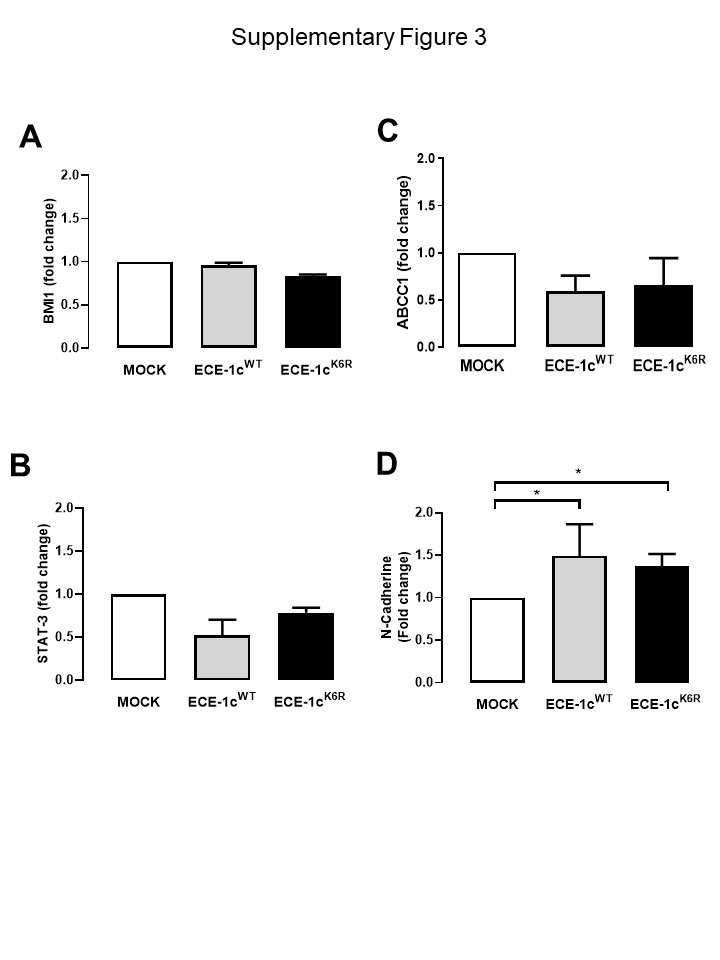

Supplement: Supplementary file 3 — Supplementary Figure 3. Transcript levels of BMI-1, Stat-3, ABCC1 and N-cadherin in ECE-1c-expressing A549 lung cancer cells. Mock, ECE-1cWT- and ECE-1cK6R-expressing A549 human lung cancer cells were grown under normal conditions for 24 h. Then, mRNA levels of BMI-1 (A), Stat-3 (B), ABCC1 (C) and N-cadherin (D) were quantified by RT-qPCR. Values were plotted as mean ± SE from at least three independent experiments performed in triplicate. *P ≤ 0.05. [file 40659_2024_551_MOESM3_ESM.tif]

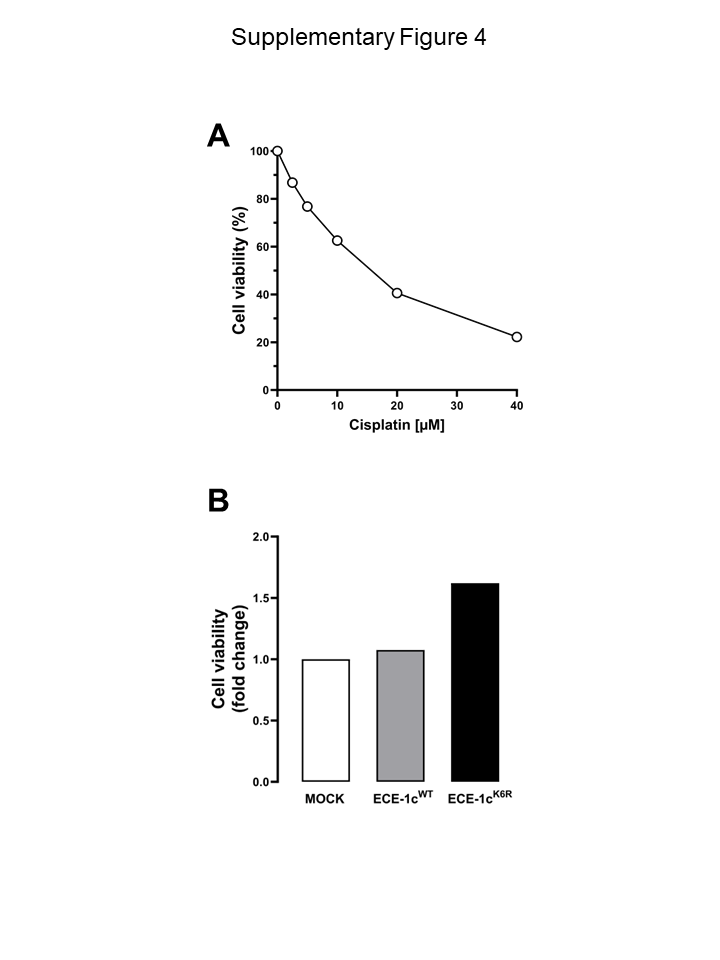

Supplement: Supplementary file 4 — Supplementary Figure 4. ECE-1cK6R expression leads to enhanced drug resistance in H1299 lung cancer cells. (A) Dose-response analysis performed by incubating non-transduced cells with 0, 2.5, 5, 10, 20 and 40 μM cisplatin for 48 h. Viability was measured by the MTS assay. DC50 was calculated in 14.4 μM cisplatin. (B) Mock, ECE-1cWT- and ECE-1cK6R-expressing cells were grown under normal conditions in presence of 10 μM cisplatin for 48 h, and viability was measured as in A. Mean values were plotted from two independent experiments performed in triplicate. [file 40659_2024_551_MOESM4_ESM.tif]

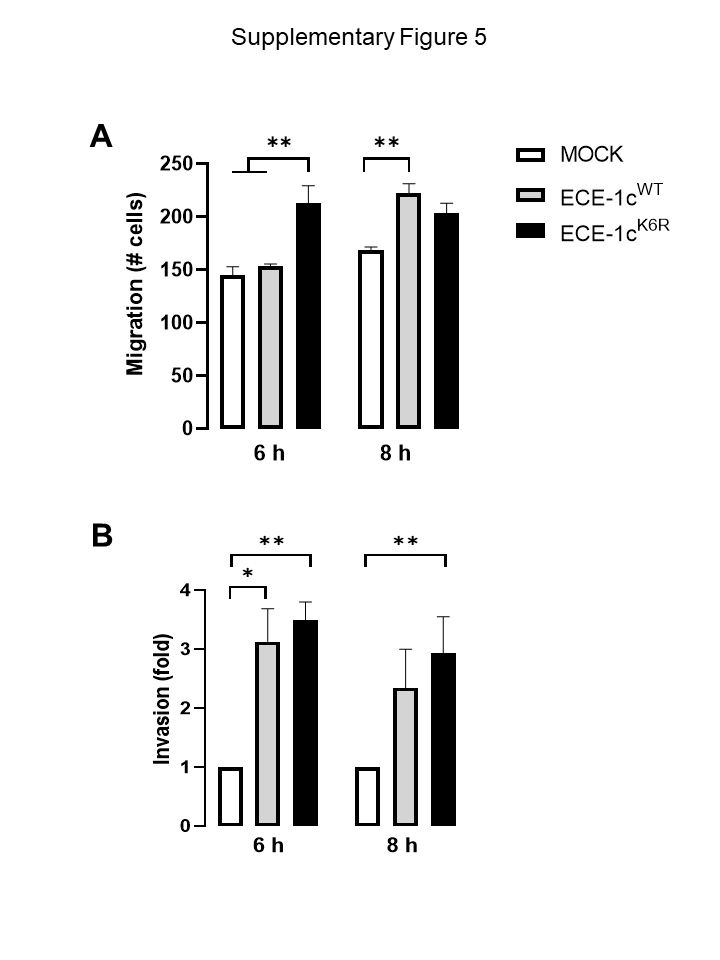

Supplement: Supplementary file 5 — Supplementary Figure 5. Migration and invasion are enhanced in ECE-1cK6R-expressing H1299 lung cancer cells. (A) Migration capacity of mock, ECE-1cWT- and ECE-1cK6R-expressing cells was evaluated in transwell chambers for 6 and 8 h. (B) Invasion capacity of cells as in A was evaluated by a Matrigel assay at 6 and 8 h. Cells were counted for each cell clone and values were plotted as mean ± SE from three independent experiments performed in triplicate. *P ≤ 0.05, **P ≤ 0.01. [file 40659_2024_551_MOESM5_ESM.tif]
